# Socioeconomic gradient in physical activity: findings from the PERSIAN cohort study

**DOI:** 10.1186/s12889-019-7715-z

**Published:** 2019-10-21

**Authors:** Ali Kazemi Karyani, Behzad Karmi Matin, Shahin Soltani, Satar Rezaei, Moslem Soofi, Yahya Salimi, Mehdi Moradinazar, Mohammad Hajizadeh, Yahya Pasdar, Behrooz Hamzeh, Loghman Barzegar, Ali Akbar Haghdoost, Reza Malekzadeh, Hossein Poustchi, Zahra Mohammadi, Elnaz Faramarzi, Ali Reza Safarpour, Farhad Pourfarzi, Mahmood Moosazadeh, Azim Nejatizadeh, Mojtaba Farjam, Davoud Vahabzadeh, Ali Ahmadi, Fereshteh Ghorat, Jafar Ahmadi, Fariborz Mansour-Ghanaei, Mohammad Reza Mirjalili, Saeid Eslami, Najmeh Maharlouei, Seyed Mehdi Tabatabaei, Sara Sarvandian, Farid Najafi

**Affiliations:** 1 Research Center for Environmental Determinants of Health (RCEDH), Health Institute, Kermanshah University of Medical Sciences, Kermanshah, Iran; 2 Social Development and Health Promotion Research Center, Health Institute, Kermanshah University of Medical Sciences, Kermanshah, Iran; 3 School of Health Administration, Faculty of Health, Dalhousie University, Halifax, Canada; 4 Nutritional Sciences Department, School of Public Health, Kermanshah University of Medical Sciences, Kermanshah, Iran; 5 Department of Public Health, Kermanshah University of Medical Sciences, Kermanshah, Iran; 6 Modeling in Health Research Center, Institute for Future Studies in Health, Kerman University of Medical Sciences, Kerman, Iran; 7 Liver and Pancreatobiliary Disease Research Center, Digestive Diseases Research Institute, Tehran University of Medical Sciences, Tehran, Iran; 8 Liver, Pancreatic, and Biliary Diseases Research Center, Digestive Diseases Research Institute, Tehran University of Medical Sciences, Tehran, Iran; 9 Liver and Gastrointestinal Diseases Research center, Tabriz University of Medical Sciences, Tabriz, Iran; 10 Gastroenterohepatology Research Center, Shiraz University of Medical Sciences, Shiraz, Iran; 11 Digestive Disease Research Center, Ardabil University of Medical Sciences, Ardabil, Iran; 12 Health Sciences Research center, Addiction Institute, Mazandaran University of Medical Sciences, Sari, Iran; 13 Molecular Medicine Research Center, Hormozgan University of Medical Sciences, Bandar Abbas, Iran; 14 Noncommunicable Diseases Research Center, Fasa University of Medical Sciences, Fasa, Iran; 15 Social Determinants of Health Research Center, Urmia University of Medical Sciences, Urmia, Iran; 16 Modeling in Health Research Center, Shahrekord University of Medical Sciences, Shahrekord, Iran; 17 Traditional and Complementary Medicine Research Center, Sabzevar University of Medical Sciences, Sabzevar, Iran; 18 Department of Radiology, Medical School, Rafsanjan University of Medical Sciences, Rafsanjan, Iran; 19 Gastrointestinal and Liver Diseases Research Center, Guilan University of Medical Sciences, Rasht, Iran; 20 Shahid Sadoughi University of Medical Sciences, Yazd, Iran; 21 Pharmaceutical Research Center, Pharmaceutical Research Institute, Mashhad University of Medical Sciences, Mashhad, Iran; 22 Department of Medical Informatics, Faculty of Medicine, Mashhad University of Medical Sciences, Mashhad, Iran; 23 Department of Medical Informatics, University of Amsterdam, Amsterdam, The Netherlands; 24 Health Policy Research Center, Shiraz University of Medical Sciences, Shiraz, Iran; 25 Health Promotion Research Center, Zahedan University of Medical Sciences, Zahedan, Iran; 26 Department of Biostatistics and Epidemiology, School of Public Health, Ahvaz Jundishapur University of Medical Sciences, Ahvaz, Iran

**Keywords:** Inequality, Physical activity, Socioeconomic status, Iran, PERSIAN

## Abstract

**Background:**

The level of socioeconomic-related inequality in physical activity in Iran is largely unknown. This study investigates socioeconomic-related inequality in poor-physical activity (PPA) among Iranian adults.

**Methods:**

A total of 129,257 adult participants enrolled in the PERSIAN (Prospective Epidemiological Research Studies in IrAN) Cohort were included in this study. Physical activity of adults was measured using metabolic equivalent rates (METs). Physical activity less than 41 METs/hour/day was considered PPA. The Concentration index (C) was used to quantify socioeconomic-related inequality in PPA. Moreover, the C was decomposed to identify the relative contribution of explanatory variables to inequality in PPA.

**Results:**

There were significant regional variations in physical activity level among Iranian adults (29.8–76.5%). The positive value of C (0.098, 95% CI = 0.092 to 0.104) suggested that the higher concentration of PPA among higher socioeconomic status (SES) adults in Iran which was consistently observed in all cohort sites.

**Conclusions:**

The higher prevalence of PPA among Iranian adults, especially, women and older adults, warrant further public health attention. Since PPA is concentrated more among the high-SES population in Iran, strategies for the promotion of physical activity should focus more on economically well-off population.

## Background

Insufficient physical activity is one of the most important risk factors for chronic diseases worldwide. Globally, physical inactivity accounts for 10% of breast and colon cancers, 7% of type two diabetes and 6% of the burden of disease from ischemic heart disease [1]. Diabetes mellitus along with hypertension are major risk factors for cardiovascular diseases. The cost of treating these risk factors secondary to lack of physical activities are causing severe economic strain to many economies [2–4]. It is estimated that 5.3 million premature deaths in 2008 were caused by lack of physical activity worldwide [5]. Physical inactivity can lead to disability and exacerbate the severity of disabilities. The findings of a study indicated that inactivity led to 3% of disability-adjusted years of life lost (DALYs) in the UK 2002 and 1.1 billion of direct costs to the National Health Service (NHS) in the UK [6].

The level of physical activity is affected by various factors. Some studies note that the factors such as age, smoking status, education, body mass index (BMI), stress, gender (being female), higher educational levels, emotional distress, family satisfaction, income, diseases and depression have a negative association with the physical activity level [7]. In contrary, some work indicate the variables such as socioeconomic status (SES), access to exercise facilities, social supports, being married, positive beliefs and attitudes, sunny weather conditions, better health status have a positive impact on the physical activity level [8, 9]. The current studies highlighted socioeconomic and cultural factors as the main predictors of the level of physical activity [10].

Findings of the third national surveillance of risk factors of non-communicable diseases indicated that 40% of Iranian adults have low physical activity and physical inactivity is more prevalent in women and older age adults [11]. Also, the prevalence of physical inactivity has been increasing trend during the recent years [12].

In Iran, several studies have been conducted to determine the predictors of the level of physical activity. The existing studies examined the level of physical activity among different groups including children, adolescents, elderlies, pregnant women, students, employees and patients with chronic diseases. These studies revealed age, gender, place of residency, socioeconomic status, having breakfast, nutritional behaviors, lifestyle, pregnancy, education, physical activity training, marital status (married couples), social relationships, mental and intellectual disorders, body function, physical self-perception and communication technologies as the main determinants of physical activity level in Iran [13–15].

Types and levels of physical activity vary among socioeconomic groups. People in higher SES groups have higher levels of physical activity and lower SES is associated with more occupational-related activities [16]. Therefore, there are a contradiction about the total effect of socioeconomic variables on PPA. On the other hand, socioeconomic-related inequality in physical activity can be explained due to differences in health indicators such as overweight and obesity that are the main predictors of mortality and morbidity [17]. Also, physical activity is multifactorial and several demographic and socioeconomic factors have an effect on the prediction of the level of physical activity in the population [18]. In addition, the availability of related evidence about the contribution of the main factors to the health related indicators, such as physical activity, is critical for policy making in this field [19].

Although the available studies examined factors associated with physical activity level in Iran, these studies did not explain the variability of physical activity in Iran sufficiently. Thus, in this study, we aimed to investigate the variability of poor physical activity (PPA) in Iranian adult population to identify the main factors (including SES) contributing to the inequality in PPA in Iran.

## Methods

### Data source and variables

In this analysis, we extracted and merged the required data from the Prospective Epidemiologic Research Studies in IrAN (PERSIAN), in 14 provinces in Iran, since 2014. The PERSIAN cohort is a cohort study that has different studying sites around Iran. Because of the coordination among these cohorts, the data collection tools and their definitions were comparable; therefore, we could compile their datasets with minimum conflicts. Some Iran provinces have more than one cohort sites. Therefore, data from these sites were merged together. The Yasuj cohort was excluded from the study because this cohort was in the data collection phase at the time of the current study. Appendix shows the characteristics of cohort sites in Iran.

After excluding the subjects with the missing values in the variables included in the study, a total of 129,257 adults, aged 35 years and above, from 14 provinces of Iran were included in the analysis. The data of the collated using a valid and reliable questionnaire that was designed to collect data from participant in all PERSIAN cohort sites. Participants were invited to one of the cohort sites and interviewed with a trained person. Detailed information on the PERSIAN Cohort Study can be found elsewhere [20, 21].

The outcome variable was PPA which was measured using metabolic equivalent rates (METs) of self-reported daily activities of participants of PERSIAN cohort using the questionnaire. A MET is equal to resting metabolic rate, the amount of oxygen consumed at rest that is about 3.5 ml 0_2_/kg/min. As four METs requires 16 ml 0_2_/kg/min [22] MET of each activity were extracted using compendium of physical activities [23]. According to the mean MET rates of participants (41 METs/hour/day), participants with less than 41 METs/hour/day were defined as individuals with PPA level.

Explanatory variables of this study were sex, age, marital status, smoking status, hookah smoking, alcohol drinking, BMI, place of residency (province), and socioeconomic status that were included based on previous literatures in this field [13–15, 24]. Included data was related to recruitment phase of the Persian cohort sites. The BMI of participants was calculated as weight in kilograms divided by square of height in meters. As per World Health Organization (WHO) classification, individuals with the BMI values of less than 18.5, 18.5–24.9, 25–29.9 and 30 kg/m2 and above were considered as underweight, normal, overweight and obese, respectively [25]. Smoking status evaluated based on the current National Health Interview Survey (NHIS) smoking definition, which screens for lifetime smoking ≥100 cigarettes. While the current smokers are those who smoke on a regular basis, the former smokers are those who quit cigarette and/or tobacco use [26]. Use of hookah, alcohol consumption and drug abuse were other variables included in the study. Age categorized into four groups of 35–44, 45–54, 55–64 and ≥ 65 years old.

### Statistical analysis

#### Socioeconomic status index

A principal component analysis (PCA) method was used to construct a socioeconomic status (SES) index of respondents in the Cohort Study [27]. Available information on infrastructure facilities (source of drinking water, sanitation facility), housing condition (e.g. the number of rooms, type of home ownership) and ownership of a range of durable assets (e.g. dishwasher, car, television), and education level in the dataset was used in the construction of SES variable for each participants. Participants of the study categorized into five SES quintiles, from the lowest (1st quintile) to the highest (5th quintile) SES groups.

#### Measuring socioeconomic-related inequality in PPA

The Concentration index (C) approach was used to measure socioeconomic-related inequality in PPA among adults in Iran. The C is based on a concentration curve which plots the cumulative proportion of population ranked according to their SES on the x-axes against the cumulative proportion of health outcome on the y-axes. Twice the area between the line of perfect equality (45-degree line) and the concentration curve is defined as the C. The C ranges between − 1 and + 1. A positive (negative) value of the C indicates that PPA is concentrated among the groups with high (low) SES groups. If the value of the C equal to zero, it indicates that PPA is equally distributed among the different socioeconomic groups. The following formula can be used to measure the C [28]:

1 $$ C=\frac{2\ast \mathit{\operatorname{cov}}\left({y}_i\ {r}_i\right)\ }{\mu } $$

Where *y*_*i*_ is the dependent variable (i.e. PPA) for the participant *i*, *r*_*i*_ is the fractional rank of participant *i* in the SES distribution, and *μ* is the mean of the dependent variable. Since PPA is a binary variable, we used normalized the C as per Wagstaff’s suggestion [29] by multiplying the C by 1/1 − *μ*.

#### Decomposing socioeconomic-related inequality in PPA

The C was decomposed to identify the contribution of explanatory variables to the observed socioeconomic-related inequality in PPA among adults in Iran [30]. If we have a regression model that links PPA to a set of *k* explanatory variables (sex, age, marital status, smoking status, drugs use, hookah smoking, BMI, place of residence (province)) and SES as:

2 $$ y=a+\sum \limits_k{\beta}_k{x}_k+\varepsilon, $$

The C for *y* can be decomposed as follows:

3 $$ C=\sum \limits_k\left(\frac{\beta_k{\overline{x}}_k}{\mu}\right){C}_k+{GC}_{\varepsilon }/\mu . $$

Where *β*_*k*_ is the coefficient of each explanatory variable (in here marginal effect of each explanatory variable calculated from the logit model), ${\overline{x}}_k$ is the mean of each explanatory variable, *C*_*k*_ is the concentration index for each independent variable, *GC*_*ε*_ is the generalized concentration index for *ε*. The $\sum \limits_k\left(\ \frac{\beta_k{\overline{x}}_k}{\mu}\right){C}_k$ component in Eq. 3 indicates the proportion of the C explained by the systematic variation of the explanatory variables across socioeconomic groups. The negative (positive) contribution of an independent variable suggests that socioeconomic distribution of that variable and its relation with PPA lead to a lower likelihood of PPA among the poor (the rich). The $\frac{G{C}_{\varepsilon }}{\mu }$ component Eq. 3 formula specifies the proportion of socioeconomic-related inequality which is not explained by the explanatory variables included in the model. Similarly normalized concentration index, *NC*, can be decomposed using the following formula [29]:

4 $$ NC=\frac{C}{1-\mu }=\frac{\sum \limits_k\left(\frac{\beta_k{\overline{x}}_k}{\mu}\right){C}_k}{1-\mu }+\frac{G{C}_{\varepsilon }/\mu }{1-\mu } $$

All the analyses were performed by Stata software version 14.2 (StataCorp, College Station, TX, USA). The geographical maps were depicted by ArcGIS software Version 10.6.1.

## Results

**Fig. 1:**
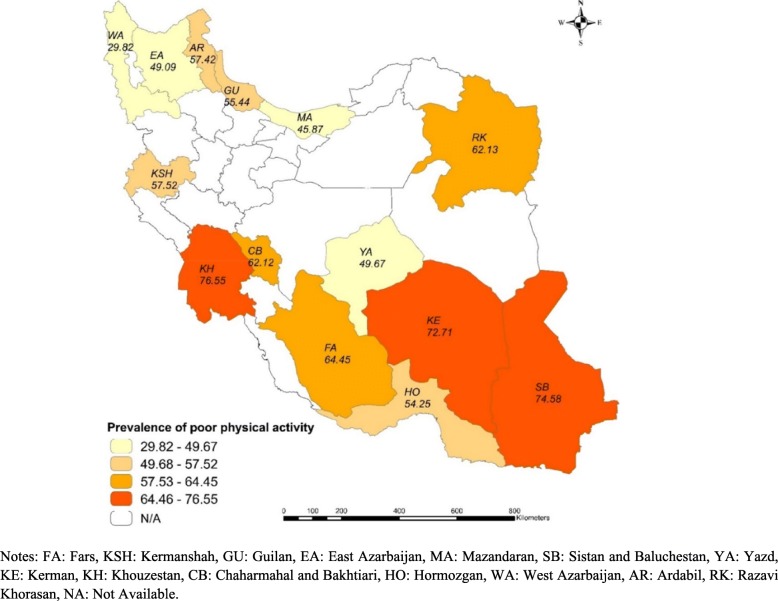
The prevalence of poor physical activity among Iranian provinces

Table 1 shows descriptive statistics of participants of the PERSIAN cohort by PPA status. The descriptive results suggest that approximately 60% of the total participants had PPA. The population of Khouzestan province cohort had the highest (76.5%) and West Azerbaijan cohort’ population had the lowest (29.8%) prevalence of PPA among 14 Iranian provinces included in the study. The prevalence of PPA among women was higher than males (64.5% vs. 52.8%). Older adults had the highest prevalence of PPA (70%). The prevalence of PPA was 58% among married individuals whereas the corresponding prevalence was 72% for divorced/separated participants. Those who were had the highest prevalence of PPA (69.6%). The proportion of PPA among current and former smokers was slightly lower than their non-smoker counterparts. The distribution of participants among SES quintiles is almost the same (about 20% in each quintile). Participants in the highest SES group had the highest prevalence of PPA (67.7%) followed by fourth quintile (60%). Figure 1 displays the prevalence of PPA among 14 provinces included in the study. As shown in the figure, the north and north eastern provinces had a lower prevalence for PPA.

**Table 1:** Characteristics of participants of Persian Cohort Study by poor physical activity status

Variables	Total	PPA
	N (%)	N (%)
Sex		
Male	57,614 (44.57)	30,389 (52.75)
Female	71,643 (55.43)	46,187 (64.47)
Age groups (years)		
35–44	45,809 (35.44)	26,272 (57.35)
45–54	43,481 (33.64)	24,941 (57.36)
55–64	31,573 (24.43)	19,521 (61.83)
> =65	8394 (6.49)	5842 (69.60)
Marital status		
Single	2910 (2.25)	1930 (66.32)
Married	117,521 (90.92)	68,286 (58.11)
Widowed and divorced	8826 (6.83)	6360 (72.06)
Smoking status		
No	101,136 (78.24)	61,212 (60.52)
Current	18,115 (14.01)	9606 (53.03)
Former	10,006 (7.74)	5758 (57.55)
Hookah smoking		
No	114,949 (88.93)	68,318 (59.43)
Yes	14,308 (11.07)	8258 (57.72)
Drug abuse		
No	113,812 (88.05)	67,742 (59.52)
Yes	15,445 (11.95)	8834 (57.20)
Alcohol drinking		
No	117,559 (90.95)	70,287 (59.79)
Yes	11,698 (9.05)	6289 (53.76)
BMI		
Underweight	2561 (1.98)	1293 (50.49)
Normal weight	34,671 (26.82)	18,478 (53.30)
Overweight	52,688 (40.76)	31,396 (59.59)
Obese	39,337 (30.43)	25,409 (64.59)
Provinces		
Fars (FA)	22,939 (17.75)	14,784 (64.45)
Khouzestan (KH)	8991 (6.96)	6883 (76.55)
Sistan and Baluchestan (SB)	8152 (6.31)	6080 (74.58)
Kerman (KE)	9885 (7.65)	7187 (72.71)
Razavi Khorasan (RK)	2868 (2.22)	1782 (62.13)
Chaharmahal and Bakhtiari (CB)	6655 (5.15)	4134 (62.12)
Kermanshah (KSH)	10,040 (7.77)	5775 (57.52)
Ardabil (AR)	8178 (6.33)	4696 (57.42)
Guilan (GU)	10,494 (8.12)	5818 (55.44)
Hormozgan (HO)	3285 (2.54)	1782 (54.25)
Yazd (YA)	9388 (7.26)	4663 (49.67)
East Azerbaijan (EA)	14,958 (11.57)	7343 (49.09)
Mazandaran (MA)	10,252 (7.93)	4703 (45.87)
West Azerbaijan (WA)	3172 (2.54)	946 (29.82)
Socioeconomic Status (SES)		
1st quintile (lowest)	25,995 (20.11)	14,344 (55.18)
2nd quintile	25,901 (20.04)	14,402 (55.60)
3rd quintile	25,819 (19.97)	14,887 (57.66)
4th quintile	25,778 (19.94)	15,490 (60.09)
5th quintile (highest)	25,764 (19.93)	17,453 (67.74)
Total	129,257 (100)	76,576 (59.24)

Table 2 contains the estimated values of the *NC* for all included cohort. The statistically significant positive value of the *NC* for all included populations (*NC* =0.098, 95% confidence interval [CI] = 0.0917 to 0.104) indicated a higher concentration of PPA among the high-SES adults. The higher prevalance of PPA among the high-SES participants also was observed within the provinces included in the study. The lowest and highest significant socioeconomic-related inequality in PPA were found in cohorts of Fars (*NC* = 0.041) and Mazandaran (*NC* =0.360), respectively. The values of the *NC* for all cohorts, except Yazd and Hormozgan, were statistically significant (*p* < 0.01).

**Table 2:** The normalized concentration index for PPA in Iran and across its provinces

Sample	NC	95% Confidence Interval	*P*-value
FA	0.041	0.025 to 0.057	< 0.001
KSH	0.204	0.182 to 0.226	< 0.001
GU	0.202	0.18 to 0.224	< 0.001
EA	0.102	0.084 to 0.12	< 0.001
MA	0.360	0.338 to 0.382	< 0.001
SB	0.150	0.123 to 0.177	< 0.001
YA	0.020	−0.004 to 0.044	0.087
KE	0.074	0.049 to 0.099	< 0.001
KH	0.042	0.015 to 0.069	0.003
CB	0.046	0.019 to 0.073	0.001
HO	0.017	−0.022 to 0.056	0.401
WA	0.106	0.063 to 0.149	< 0.001
AR	0.116	0.092 to 0.14	< 0.001
RK	0.126	0.091 to 0.161	< 0.001
Total	0.098	0.092 to 0.104	< 0.001

*FA* Fars, *KSH* Kermanshah, *GU* Guilan, *EA* East Azarbaijan, *MA* Mazandaran, *SB* Sistan and Baluchestan, *YA* Yazd, *KE* Kerman, *KH* Khouzestan, *CB* Chaharmahal and Bakhtiari, *HO* Hormozgan, *WA* West Azarbaijan, *AR* Ardabil, *RK* Razavi Khorasan, *PPA* Poor Physical Activity, *NC* Normalized Concentration Index

**Fig. 2:**
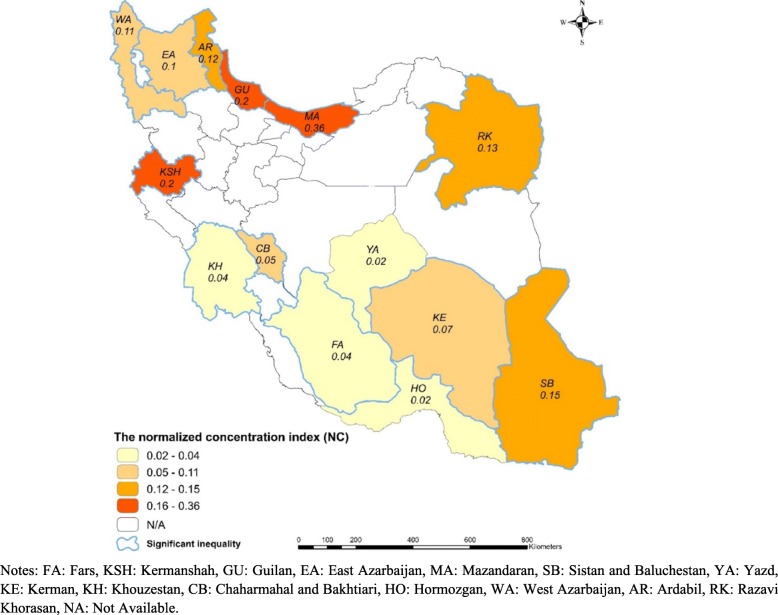
Socioeconomic-related inequality in physical activity among Iranian provinces

As illustrated in Fig. 2, the lowest and the highest socioeconomic-related inequality in PPA was observed in the cohort population of the northern and southern provinces of Iran, respectively.

Table 3 presents the results of the decomposition of socioeconomic-related inequality in PPA. The table reports the elasticity of PPA with respect to each explanatory variable, the C for each explanatory variables (*C*_*k*_), absolute and percentage contribution of each explanatory variable to the *NC*.

**Table 3:** Decomposition results of socioeconomic-related inequality in poor physical activity in Iran

Variables	Marginal Effects	Elasticity	The concentration Index (*C*_*k*_)	Absolute Contribution	Percentage contribution	Summed Percentage Contribution
Sex (ref: male)						
Female	0.14	0.131	−0.067	−0.02	−21.83	−21.83
Age groups (ref: 35–44 years)						
45–54	0.01	0.005	0.026	0.0003	0.30	−25.08
55–64	0.08	0.033	−0.125	− 0.01	−10.34	
> =65	0.19	0.020	−0.295	−0.01	−15.04	
Marital status (ref: single)						
Married	−0.10	−0.152	0.024	−0.01	−9.19	−8.21
Widowed and divorced	−0.01	−0.001	− 0.288	0.00	0.97	
Smoking status (ref: never smoker)						
Current smoker	0.03	0.006	−0.015	− 0.00024	−0.24	− 0.19
Former smoker	0.02	0.003	0.006	0.00005	0.05	
Drug abuse (ref: no)						
Yes	0.01	0.002	−0.030	−0.00017	−0.17	− 0.17
Alcohol drinking (ref: no)						
Yes	0.01	0.002	0.172	0.00091	0.93	0.93
BMI (ref: Normal weight)						
Underweight	−0.04	−0.001	−0.300	0.001	1.01	10.04
Overweight	0.06	0.044	0.048	0.01	5.22	
Obese	0.11	0.057	0.027	0.004	3.80	
Province (ref: Fars)						
KSH	−0.10	−0.013	−0.07	0.002	2.07	−40.72
GU	−0.13	−0.018	− 0.16	0.01	6.89	
EA	−0.22	−0.043	− 0.03	0.003	2.95	
MA	−0.29	−0.039	0.13	−0.01	−12.84	
SB	0.04	0.004	0.05	0.001	0.51	
YA	−0.23	−0.028	0.16	−0.01	−11.56	
KE	−0.01	−0.002	0.31	−0.001	−1.20	
KH	0.08	0.009	−0.16	−0.004	−3.59	
CB	−0.15	−0.013	0.45	−0.01	−14.76	
HO	−0.15	−0.006	− 0.07	0.00	1.04	
WA	−0.41	−0.017	− 0.20	0.01	8.49	
AR	−0.17	−0.019	0.21	−0.01	−9.89	
RK	−0.16	−0.006	0.59	−0.01	−8.83	
Socioeconomic status (SES) (ref: 1st quintile)						
2nd quintile	0.06	0.019	−0.397	−0.02	−19.00	205.32
3rd quintile	0.10	0.034	0.003	0.00	0.23	
4th quintile	0.15	0.052	0.402	0.05	52.17	
5th quintile (highest)	0.26	0.086	0.801	0.17	171.91	
Explained				0.12		120.09
Residuals				−0.02		−20.09
Total NC				0.10		100.00

Based on the results of the marginal effects, compared to men, women had 14% higher probability of having PPA. Age had a positive correlation with PPA and older adults (> = 65 years old) had a 19% higher probability for PPA than participants aged 35–44 years old. The probability of PPA was 10% lower among married participants than their single counterparts. There was a positive association between smoking and PPA among PERSIAN cohort participants. Obese adults had a higher probability of having PPA than normal weight adults. There was a positive correlation between SES and PPA among adults. The probability of PPA among the highest SES quintile group was 26% greater than the lowest SES quintile group. The C for independent variables (*C*_*k*_) suggested that women, older age groups, being a widow/ divorced, smoker, drug users and underweight were more concentrated among the poor people. However, alcohol drinking, overweight and obesity were more concentrated among the rich people.

The results of the decomposition suggested SES, itself, was the main contributor to the concentration of PPA among high-SES adults in Iran. BMI was another factor that increased the concentration of PPA among the high-SES individuals. In contrast, place of residence (province), age groups, sex, marital status made negative contributions (i.e., increase the concentration of PPA among the low-SES adults) to socioeconomic-related inequality in PPA.

## Discussion

This is one of the unique national studies which investigated socioeconomic inequalities in poor physical activity among Iranian adults. The positive value of the estimated *NC* for all cohort sites indicated that higher SES adults tend to have higher PPA than their lower SES counterparts. SES is the largest contributor to socioeconomic-related inequality in PPA. In contrast to our findings, other studies documented a positive association between an individual’s SES and the level of physical activity. For example, the findings of a study by Meltzer and Jena [31] indicated that people in the highest income group were more likely to have higher energy expenditure and exercise intensity compared to the lowest income group. Humphreys and Ruseski [32] also showed that individuals in higher income groups tended to have more diverse types of physical activity than lower income groups.

The results showed that there is a relationship between income and physical activity, but the relation is gender-specific and depends on how researchers measure that. For example, The findings of Kari et al. showed that people with higher income were more likely to have higher physical activity than those with lower income in general, but in more detail the relationship was positive for women and negative or non–existing for men so that women indicated a positive association between higher income and aerobic steps compared to men [33]. Similarly, in a national health survey study in the US from 2007 to 2016, Armstrong et al. found that the race/ethnicity and low income have a positive association with physical activity in the most groups [34]. However, some studies found no or a negative association between socioeconomic factors and physical activity. For example, McAuley et al. in the US found no association between variables like age, gender, ethnicity, marital status, education and income with physical activity [35]. On the other hand, Finkelstein et al. indicated a negative relationship between education and income with physical activity [36]. It should be noted studies show mixed results with socioeconomic determinants of physical activity internationally [10]. Different sociocultural contexts and objective measures may be a reason for the variation in the literature.

It must be noted that the association between SES and physical activities can be more complicated than expected. Although higher SES provides more opportunities for being physically active, it may also decrease the amount of time spent on physical activity due to the opportunity cost of time spent on physical activity [32, 37]. Accordingly, some studies revealed that people with higher income may have more intense physical exercise and less general attention to exercise [31, 38, 39]. Similarly, Ruham’s (2000) findings illustrated that unemployed compared to employed individuals tended to have higher levels of physical activity [40].

Our findings indicated that place of residence (province), age, and sex were the main negative contributors of SES-related inequality among included population in this study. Accordingly, the variation of participants by those variables decreased the concentration of PPA among well-off adults. We found that the proportion of PPA among smoker participants was slightly lower than non-smokers. But, the results of marginal effects analysis, that adjusted the effects of smoking status on the PPA, indicated a direct association between smoking and physical inactivity. Similarly, other study reported a negative correlation between smoking and poor physical activity in Iran [41].

The results suggested wide variation in the prevalence and socioeconomic-related inequality in PPA among cohorts of PERSIAN. While population of the cohorts of Mazandaran, Guilan and Kermanshah (located in north and west of Iran) had the highest value of the *NC*, the cohort provinces of Hormozgan (in the south), Yazd (in the center) and Fars (located in south and center of Iran) had the lowest value of the *NC*. On the other hand, the findings revealed that Kermanshah and Guilan provinces’ cohort population were more concentrated among the low SES and population of Yazd and Fars provinces’ cohorts were more concentrated among the high SES. Therefore, it seems that the cohort population with lower SES had more probability to have a higher level of inequality in PPA, vice versa. However, there was no significant difference among cohort studies for prevalence of overweight and obesity.

While environmental and regional climate variation among the provinces in Iran may have affected the physical activity levels of adults living in different provinces differently, cultural diversities between the provinces may have contributed to the observed differences in socioeconomic-related inequality in PPA among provinces in Iran.

In addition to the higher prevalence of PPA among high-SES adults, the results of our study highlighted a higher prevalence of PPA among women compared to men (64.5% vs. 52.8%). Other studies in Iran also indicate that women significantly have less physical activity than men [42, 43]. Women face several cultural barriers to participate in physical activities in Iran. Studies in Iran demonstrated cultural believes that restrict access to some sports locations and participate in particular forms of physical activities in Iran [44, 45]. Other studies indicated a lack of confidence and self-consciousness as the main factors that limit the levels of physical activity for women [46]. The results also suggested that single adults were more likely to report PPA than married adults. As discussed in the literature, being in a stable relationship improves healthy behavior of adults [47, 48], which, in turn, leads to a positive effect on health outcomes [48, 49]. Participation in physical activity remains lower among middle and older age groups in comparisons to the younger age groups. Studies in Iran showed that factors such as loneliness, depression, lack of enjoyment, disability and chronic diseases as the most important barriers to participation in physical activity for older people [50]. We also found a positive association between BMI and PPA. This is consistent with the current literature [51], which shows that overweight and obesity independently are associated with PPA.

This study used national data from PERSIAN cohort studies to investigate the prevalence and socioeconomic-inequality in PPA in Iran provinces. The results provide new evidence about inequality in PPA and its contributors in the country. However, this study is subject to some limitations. First, we analyzed PPA in provinces using the data from PERSIAN cohort studies which might not be representative of the whole country and even whole province. However, the PERSIAN cohort is the largest cohort study including more than 120,000 populations from different ethnicity with the same methods and questionnaires and this in turn increase the validity of our study. Second, the PPA was measured using self-reported daily activities. A potential for recall bias exists for self-reported daily activities. However, any recall bias regarding the level of PPA is high likely to be non-differential and therefore we expect to see some underestimation of the effect size. Third, causal inferences cannot be made because of the cross-sectional nature of our study design.

## Conclusions

We found that PPA is concentrated among high-SES adults in Iran and SES, itself, is the largest contributor to the observed inequality. Thus, strategies for the promotion of physical activity in Iran should focus more on economically well-off population.

## Data Availability

The datasets generated and/or analyzed during the current study are not publicly available because some results are still being analyzed. Data can be obtained from the corresponding author on reasonable request after permission of the central team of PERSIAN Cohort study.

## Appendix

**Table 4:** The cohort population in the study provinces

Row	Province	^a^Population	Cohort site	^a^Population	Cohort population	Main Ethnicities
1	Ardabil	1,270,420	Ardabil	529,374	8192	Turk
2	Chaharmahal and Bakhtiari	947,763	Sharekord	93,104	6664	Lor
3	East Azerbaijan	3,909,652	Khameneh	3056	14,978	Turk, Azari
4	Fars	4,851,274	Kavar	31,711	2244	Fars (Persian), Turk
			Kharameh	18,477	10,662	Fars (Persian), Arab
			Fasa	110,825	10,113	Fars (Persian), Arab and Turk
5	Guilan	2,530,696	Some’e Sara	58,658	10,511	Gilaki
6	Hormozgan	1,776,415	Bandare Kong	19,213	3570	Arab
7	Kerman	3,164,718	Rafsanjan	161,909	9982	Fars (Persian)
8	Kermanshah	1,952,434	Ravansar	47,657	10,077	Kurd
9	Khouzestan	4,710,506	Hoveizeh	19,481	9156	Arab
10	Mazandaran	3,283,582	Sari	309,820	10,253	Tabari
11	Razavi Khorasan	6,434,501	Mashhad	3,001,184	2189	Fars (Persian)
			Sabzevar	243,700	784	Fars (Persian)
12	Sistan and Balouchestan	2,775,014	Zahedan	587,730	8318	Balouch
13	West Azerbaijan	3,265,219	Ghoushchi	2787	3662	Turk, Azari
14	Yazd	1,138,533	Shahedieh, Yazd	18,309	9901	Fars (Persian)

^a^The population is according to Iranian Population and Housing Census in 2016 [40]

## Abbreviations

95% CI: 95% Confidence Intervals
BMI: Body Mass Index
C: Concentration Index
DALYs: Disability-Adjusted Years of Life Lost
METs: Metabolic Equivalent Rates
NC: Normalized Concentration Index
NHIS: National Health Interview Survey
NHS: National Health Service
PCA: Principal Component Analysis
PERSIAN: Prospective Epidemiologic Research Studies in IrAN
PPA: Poor-Physical Activity
SES: Socioeconomic Status
vs: versus
WHO: World Health Organization
