# Correction: Socioeconomic gradient in physical activity: findings from the PERSIAN cohort study

**DOI:** 10.1186/s12889-022-14049-0

**Published:** 2022-09-09

**Authors:** Ali Kazemi Karyani, Behzad Karmi Matin, Shahin Soltani, Satar Rezaei, Moslem Soofi, Yahya Salimi, Mehdi Moradinazar, Mohammad Hajizadeh, Yahya Pasdar, Behrooz Hamzeh, Loghman Barzegar, Ali Akbar Haghdoost, Reza Malekzadeh, Hossein Poustchi, Zahra Mohammadi, Elnaz Faramarzi, Ali Reza Safarpour, Farhad Pourfarzi, Mahmood Moosazadeh, Azim Nejatizadeh, Mojtaba Farjam, Davoud Vahabzadeh, Ali Ahmadi, Fereshteh Ghorat, Jafar Ahmadi, Fariborz Mansour-Ghanaei, Mohammad Reza Mirjalili, Saeid Eslami, Najmeh Maharlouei, Seyed Mehdi Tabatabaei, Sara Sarvandian, Farid Najafi

**Affiliations:** 1 Research Center for Environmental Determinants of Health (RCEDH), Health Institute, Kermanshah University of Medical Sciences, Kermanshah, Iran; 2 Social Development and Health Promotion Research Center, Health Institute, Kermanshah University of Medical Sciences, Kermanshah, Iran; 3 School of Health Administration, Faculty of Health, Dalhousie University, Halifax, Canada; 4 Nutritional Sciences Department, School of Public Health, Kermanshah University of Medical Sciences, Kermanshah, Iran; 5 Department of Public Health, Kermanshah University of Medical Sciences, Kermanshah, Iran; 6 Modeling in Health Research Center, Institute for Future Studies in Health, Kerman University of Medical Sciences, Kerman, Iran; 7 Liver and Pancreatobiliary Disease Research Center, Digestive Diseases Research Institute, Tehran University of Medical Sciences, Tehran, Iran; 8 Liver, Pancreatic, and Biliary Diseases Research Center, Digestive Diseases Research Institute, Tehran University of Medical Sciences, Tehran, Iran; 9 Liver and Gastrointestinal Diseases Research center, Tabriz University of Medical Sciences, Tabriz, Iran; 10 Gastroenterohepatology Research Center, Shiraz University of Medical Sciences, Shiraz, Iran; 11 Digestive Disease Research Center, Ardabil University of Medical Sciences, Ardabil, Iran; 12 Health Sciences Research center, Addiction Institute, Mazandaran University of Medical Sciences, Sari, Iran; 13 Molecular Medicine Research Center, Hormozgan University of Medical Sciences, Bandar Abbas, Iran; 14 Noncommunicable Diseases Research Center, Fasa University of Medical Sciences, Fasa, Iran; 15 Social Determinants of Health Research Center, Urmia University of Medical Sciences, Urmia, Iran; 16 Modeling in Health Research Center, Shahrekord University of Medical Sciences, Shahrekord, Iran; 17 Traditional and Complementary Medicine Research Center, Sabzevar University of Medical Sciences, Sabzevar, Iran; 18 Department of Radiology, Medical School, Rafsanjan University of Medical Sciences, Rafsanjan, Iran; 19 Gastrointestinal and Liver Diseases Research Center, Guilan University of Medical Sciences, Rasht, Iran; 20 Shahid Sadoughi University of Medical Sciences, Yazd, Iran; 21 Pharmaceutical Research Center, Pharmaceutical Research Institute, Mashhad University of Medical Sciences, Mashhad, Iran; 22 Department of Medical Informatics, Faculty of Medicine, Mashhad University of Medical Sciences, Mashhad, Iran; 23 Department of Medical Informatics, University of Amsterdam, Amsterdam, The Netherlands; 24 Health Policy Research Center, Shiraz University of Medical Sciences, Shiraz, Iran; 25 Health Promotion Research Center, Zahedan University of Medical Sciences, Zahedan, Iran; 26 Department of Biostatistics and Epidemiology, School of Public Health, Ahvaz Jundishapur University of Medical Sciences, Ahvaz, Iran

**Correction: BMC Public Health 19, 1312 (2019)**

**https://doi.org/10.1186/s12889-019-7715-z**

In the original publication [1] there was an incorrect ethic approval code. The incorrect and correct code are available in this correction article, the original article has been updated.

**Incorrect Ethics approval and consent to participate**

While each cohort center received the ethical approval from local universities, for the purpose of this study and pooling all PERSIAN data, the ethics committee of Kermanshah University of Medical Sciences approved the study (IR.KUMS.REC.1397.187)

**Correct Ethics approval and consent to participate**

While each cohort center received the ethical approval from local universities, for the purpose of this study and pooling all PERSIAN data, the ethics committee of Kermanshah University of Medical Sciences approved the study **(IR.KUMS.REC.1397.866)**
